# Thyrotropin Regulates IL-6 Expression in CD34^+^ Fibrocytes: Clear Delineation of Its cAMP-Independent Actions

**DOI:** 10.1371/journal.pone.0075100

**Published:** 2013-09-25

**Authors:** Nupur Raychaudhuri, Roshini Fernando, Terry J. Smith

**Affiliations:** 1 Department of Ophthalmology and Visual Sciences, University of Michigan Medical School, Ann Arbor, Michigan, United States of America; 2 Division of Metabolism, Endocrine, and Diabetes, Department of Internal Medicine, University of Michigan Medical School, Ann Arbor, Michigan, United States of America; University Claude Bernard Lyon 1, France

## Abstract

IL-6 plays diverse roles in normal and disease-associated immunity such as that associated with Graves’ disease (GD). In that syndrome, the orbit undergoes remodeling during a process known as thyroid-associated ophthalmopathy (TAO). Recently, CD34^+^ fibrocytes were found to infiltrate the orbit in TAO where they transition into CD34^+^ orbital fibroblasts. Surprisingly, fibrocytes display high levels of functional thyrotropin receptor (TSHR), the central antigen in GD. We report here that TSH and the pathogenic anti-TSHR antibodies that drive hyperthyroidism in GD induce IL-6 expression in fibrocytes and orbital fibroblasts. Unlike TSHR signaling in thyroid epithelium, that occurring in fibrocytes is completely independent of adenylate cyclase activation and cAMP generation. Instead TSH activates PDK1 and both AKT/PKB and PKC pathways. Expression and use of PKCβII switches to that of PKCµ as fibrocytes transition to TAO orbital fibroblasts. This shift is imposed by CD34^−^ orbital fibroblasts but reverts when CD34^+^ fibroblasts are isolated. The up-regulation of IL-6 by TSH results from coordinately enhanced IL-6 gene promoter activity and increased IL-6 mRNA stability. TSH-dependent IL-6 expression requires activity at both CREB (−213 to −208 nt) and NF-κB (–78 to −62 nt) binding sites. These results provide novel insights into the molecular action of TSH and signaling downstream for TSHR in non-thyroid cells. Fibrocytes neither express adenylate cyclase nor generate cAMP and thus these findings are free from any influence of cAMP-related signaling. They identify potential therapeutic targets for TAO.

## Introduction

G protein coupled proteins, such as the thyrotropin receptor (aka thyroid stimulating hormone receptor, TSHR^2^), regulate many physiological and pathological processes [1]. Since its molecular cloning by Parmentier and colleagues [2], TSHR and its biological functions have been studied intensively. Almost everything known about its signaling derives from studies conducted on thyroid tissues and derivative epithelial cells [3]. It has recently been recognized that TSHR is expressed widely outside the thyroid gland [4]–[9]. Its role in regulating biological functions in other tissues now needs to be studied in detail. Endo *et al* [4] detected receptor mRNA in rat epididymal fat at levels approaching those in thyroid. TSH was found to elicit cAMP generation in TSHR-transfected Chinese hamster ovary cells and to regulate lipolysis in fat [4], [10]. But relatively few studies have offered details of how TSHR might function in extra-thyroidal cells.

TSHR plays a central pathogenic role in Graves’ disease (GD), an autoimmune syndrome where the thyroid gland becomes over-active and orbital connective tissue undergoes remodeling [11]. In GD, a loss of immune tolerance to that receptor and generation of activating antibodies, termed thyroid-stimulating immunoglobulins (TSI), result in hyperthyroidism [12]–[14]. It remains uncertain whether TSHR or TSI directly participates in the pathogenesis of orbital GD, a disfiguring process known as thyroid-associated ophthalmopathy (TAO) [15]–[18]. TAO is thought to result from local actions of cytokines such as IL-6 [19] which has been widely implicated in inflammation [20], [21]. IL-6 was initially described as a B cell differentiation factor that supports immunoglobulin synthesis [22]. A number of factors regulate its production, acting through both transcriptional and post-transcriptional mechanisms [20], [23]. IL-6 transcript instability is attributable, at least in part, to stem loop destabilizing (non-AU) elements [24]. TSH has been shown to upregulate IL-6 production in thyroid epithelium [25] and in non-thyroid cells, such as 3T3-L1 preadipocytes [26], [27] but the mechanisms involved and post-receptor signaling in extra-thyroidal cells remain incompletely determined.

Fibrocytes are CD45^+^CD34^+^CXCR4^+^ collagen I (Col) I^+^ pluripotent bone marrow-derived progenitor cells involved in normal wound healing, tissue remodeling, and fibrosis [28]–[30]. They present antigens, provide important co-stimulation to T cells, and produce several extracellular matrix molecules, cytokines, and other pro-inflammatory molecules [28]–[31]. They exhibit a CD45^+^CD34^+^CD31^−^CXCR4^+^Col1^+^ phenotype that allows them to be distinguished from fibroblasts and other closely related cells of the monocyte lineage [32]. Moreover, they undergo terminal differentiation into adipocytes and myofibroblasts [33]. CD34^+^ fibrocytes become far more numerous in patients with GD and infiltrate orbital and thyroid tissues in GD [34], [35]. Surprisingly, fibrocytes co-express functional “thyroid-specific” proteins, including functional thyroglobulin and TSHR [36], [37]. The levels of TSHR protein and mRNA are dramatically higher in fibrocytes than in orbital fibroblasts, including those from patients with TAO [34]–[37]. These high levels of TSHR protein as well as differences between its abundance in fibrocytes and fibroblasts have been reported earlier [36]. Moreover, when treated with TSH, fibrocytes produce inflammatory cytokines such as IL-6, IL-8, and TNF-α [34], [35]. It is thus possible that TSH-activated fibrocytes might contribute to local cytokine production.

Here, we characterize the molecular mechanisms involved in the induction of IL-6 by TSH in CD34^+^ fibrocytes and their derivative CD34^+^ orbital fibroblasts. That regulation occurs at physiologically relevant concentrations, and is mediated through both enhanced IL-6 gene promoter activity and increased IL-6 transcript stability. Unlike the actions of TSH in thyroid epithelial cells, those in fibrocytes are completely independent of cAMP generation. They are dependent on the coordinate activation of Akt and cell type-specific PKC signaling pathways which in turn are dependent upon an activation of 3-phosphoinositide dependent protein kinase-1 (PDK1). Interruption of these pathways abolishes the induction of IL-6. Thus, signaling utilized by TSHR in fibrocytes appears complex and differs substantially from that found in the thyroid.

### Experimental Procedures

#### Ethics

The activities reported have been approved by the Institutional Review Board of the University of Michigan Health Care System. The research was conducted entirely in the United States. Participants provided written consent for their participation in the study. The IRB continually monitored the conduct of this study.

#### Materials

Ficoll-Hypaque (cat #10831), GF109203X (cat #G2911), cycloheximide (cat #01810), 5,6-dichlorobenzimidazole (DRB) (cat #D1916), pyrrolidine dithiocarbamate (PDTC, cat #P8765), and 8-bromo adenosine-3′-5′-cyclic monophosphate (8 Br-cAMP) (cat #B-5386) were obtained from Sigma Aldrich (St. Louis, MO), and bovine TSH (bTSH) (cat #609385) was from Calbiochem (San Diego, CA). Synthetic oligonucleotides were generated by Invitrogen (Grand Island, NY). An inhibitor of AKT, AKTi (cat #124011), was from Calbiochem/EMD Biosciences (Gibbstown, NJ). Anti-phospho PKCµ (Ser 916, cat #916), PDK1 (cat #3062), pPDK1 (Ser 241, cat #3061), pAKT (Ser 473, cat #5171), and p-IKK (Ser176)/IKK (Ser177, cat #2078) Abs came from Cell Signaling (Boston, MA). M22 activating anti-TSHR mAb was from Kronus (Star, ID, cat #M22-5c/00-690). OSU-03012 (cat #s1106) was obtained from Selleckchem (Houston, TX). siRNA targeting AKT was from Abcam (Cambridge, MA) while those targeting CREB (cat #L-003619), PDK1 (cat #L-003017), PKCβII (cat #L-003758), PKCµ (cat #L-005028), and RelA (cat #L-003533) were from Thermo Scientific Dharmacon (Lafayette, CO). IL-6 ELISA kits were from R & D Systems (Minneapolis, MN, cat #D6050). TaqMan gene expression assay PRKD1 (GenBank X75756.1), and adenylate cyclase (cat #Hs00392747) were obtained from Applied Biosystems (Foster City, CA). Forskolin was from Active Motif (Carlsbad, CA, cat #40300).

#### Fibroblast and fibrocyte culture

Orbital fibroblasts were cultured as described previously [38] from orbital fat/connective tissue waste generated during orbital decompression for severe TAO or from normal orbital tissues. These activities have been approved by the Institutional Review Board of the University of Michigan Health Care System. Dermal fibroblasts from a cohort of TSH-resistant subjects and their unaffected relatives [39] were generously provided by Dr. Samuel Refetoff (University of Chicago). Cultures were incubated at 37°C in a 5% CO_2_ environment on poly-L-lysine-coated culture flasks and dishes. They were covered with Dulbecco’s modified Eagle’s medium (DMEM) supplemented with 2 mM glutamine, sodium pyruvate (110 mg/ml), penicillin (100 units/ml), streptomycin (100 units/ml), 4.5% glucose and 10% fetal bovine serum (FBS). They were utilized between the second and 11th passage. Their phenotype remains unchanged during this interval [38].

Fibrocytes were generated from peripheral blood mononuclear cells (PBMC) as described by Bucala *et al*. [28]. Briefly, blood was centrifuged over Histopaque-1077, following the manufacturer’s protocol. 24-well plates were inoculated with 5 × 10^6^ cells/well and covered with DMEM supplemented with 5% FBS. After 12–14 d in culture, adherent cells (<5% of starting PBMC population) were washed and removed from the substratum by scraping. Culture purity was >90% fibrocytes by fluorescence-activated cell sorter (FACS) analysis. Cell viability was >90% by trypan blue exclusion.

#### cAMP assay

cAMP levels in cell lysates were determined with an immunoassay kit (Calbiochem, San Diego, CA) following the manufacturer’s protocol.

#### Cell Sorting

Pure CD34^+^ and CD34^−^ TAO orbital fibroblast subsets were generated from parental (mixed CD34^+^ and CD34^−^) strains. They were stained with FITC-conjugated anti-human CD34 for 30 min at 4°C and sorted under sterile conditions using a BD FACS Aria III instrument (BD Biosciences, San Jose, CA).

#### RNA isolation and real-time

*PCR*-RNA was isolated using RNeasy (Qiagen, cat #74106). cDNAs were generated by reverse transcription using oligo (dT) and SuperScript III reverse transcriptase (Invitrogen). RT-PCR was performed with iQ SYBR Green Supermix (Bio-Rad, Hercules, CA). The following primers were used: RT^2^ qPCR primers for IL-6 (Ref Seq Accession # NM_000600.3), (SA Biosciences Qiagen, Cat #PPH0560B), PKCβII forward 5′-TATCTGGGATGGGGTGACAACC-3′ and reverse 5′-CGGTCGAAGTTTTCAGCGTTTC-3′; PKCβI forward 5′-TGTGATGGAGTATGTGAACGGGGG -3′ and reverse 5′-TCGAAGTTGGAGGTGTCTCGCTTG-3′ [40], Gqα forward 5′-ACAAGTACGAGCAGAACAAGGCCA-3′ and reverse 5′-AGGGCGACGAGAAACATGATGGAT-3′ [41], and Gsα forward 5′-GGCTGCCTCGGGAACAGTAAG -3′ and reverse 5′-TAATCATGCCCTATGGTGGGTG-3′ [42]. PKCµ mRNA was quantified using PRKD1 Hs00177037_m1 (Applied Biosystems Cat # L-005028). PDPK1 mRNA levels were assessed using forward 5′- CTGAGCCAGTTTGGCTGC-3′ and reverse 5′-ACGTCCTGTTAGGCGTGTGG-3′. RT-PCR reactions were performed in triplicate with glyceraldehyde-3-phosphate dehydrogenase (GAPDH) serving as the internal control on a CFX96 Real-Time PCR system (Bio-Rad). Amplification conditions consisted of initial 12 min activation at 95°C followed by 40 cycles of denaturation at 95°C for 30 s, annealing at 58°C for 30 s, and extension at 72°C for 30 s. Relative quantification of amplicons was performed using the comparative critical threshold (C_T_) method.

#### RNA stability assay

mRNA stability was determined in confluent cultures by pre-treating with bTSH (5 mIU/ml) in DMEM with 1% FBS for 12 h. Some plates were shifted to medium without TSH while others were continued in its presence. All cultures received DRB (50 µM) at time “0” and were harvested at the intervals indicated. RT-PCR was performed and data were graphed as a best fit curve.

#### Transient transfections and reporter assays

A 1171 bp fragment, spanning –1168 to +3 nt of the human IL-6 gene promoter [43] (GenBank no. NG_011640) was cloned into pGL2-basic (Promega, Madison, WI, cat #E1641). Fibroblasts were transiently transfected with 2 µg DNA using Effectene reagent (Qiagen, cat #301425). Transfection efficiency was determined by co-transfecting 0.25 µg of pRL-TK (Promega, Madison, WI, cat #E2241). Fibrocytes were transfected using Nucleofection Technology (Cologne, Germany, cat #VPA-1003, program U023) after they were detached with Accutase (Millipore, Temecula, CA, cat #SCR005). Following centrifugation at 200×*g* for 10 min, cell pellets were re-suspended in 100 µl buffer provided by the manufacturer and mixed with 2 µg of DNA. After 48 h incubations, luciferase activity was assessed in 20 µl cell extract mixed with 100 µl luciferase assay reagent (Promega, cat # E1980). Activity was measured as light output (10 s) in a Veritas Microplate Luminometer (Turner Biosystems, Sunnyvale, CA).

#### Site-directed mutagenesis

IL-6 gene promoter fragments containing mutant CREB (designated m1) and NF-κB binding sites (designated m2) were generated using the QuickChange site-directed mutagenesis kit (Stratagene, Santa Clara, CA, cat #200518) and confirmed by sequencing.

#### siRNA transfection

To knock-down expression of mRNA targets in fibroblasts and fibrocytes, specific siRNAs and their scrambled controls were transfected at a concentration of 100 nM using RNAi (Qiagen, cat #301605). Following incubations, cell lysates (15 µg protein) were subjected to Western blot analysis to verify transfection efficiency.

#### Western blot analysis

Cellular proteins were solubilized in ice-cold lysis buffer containing 0.5% Nonidet P-40, 50 mm Tris-HCl (pH 8.0), and Halt protease inhibitor mixture (Pierce, cat #87786). Nuclear proteins were prepared using the NE-PER extraction kit (Pierce, cat #78833). Cell protein was quantified (Bio-Rad, cat #500-0001), and samples were boiled in Laemmli SDS sample buffer, separated by SDS-PAGE, and transferred to Immobilon (Millipore, Temecula, CA). Membranes were incubated with primary Abs overnight at 4°C, washed, and incubated with horseradish peroxidase-labeled secondary Abs. ECL reagent (Amersham Biosciences, cat #RPN2109) was used to generate signals. Protein bands were analyzed with a densitometer and normalized against respective β-actin bands.

#### Quantification of IL-6

Confluent monolayers in 24-well plates were shifted to medium without or with bTSH (5 mU/ml) alone or in combination with the test compounds indicated in medium containing 1% FBS. Each treatment group comprised triplicate wells unless stated otherwise. Medium was collected and subjected to specific ELISA for IL-6. Samples were assayed in triplicate using a standard curve.

#### CREB and NF-κB DNA binding assays

Binding of nuclear CREB and p65/Rel A to DNA was quantified using TransAM CREB (cat #42096) and TransAM p65/Rel A kits (cat #40096), respectively (Active Motif).

#### Data analysis

Data are presented as mean ± S.D. Statistical differences were determined with the Student’s *t* test and significance considered at p<0.05.

## Results

### bTSH Induces IL-6 Protein and mRNA in Orbital Fibroblasts and Fibrocytes

**Figure 1:**
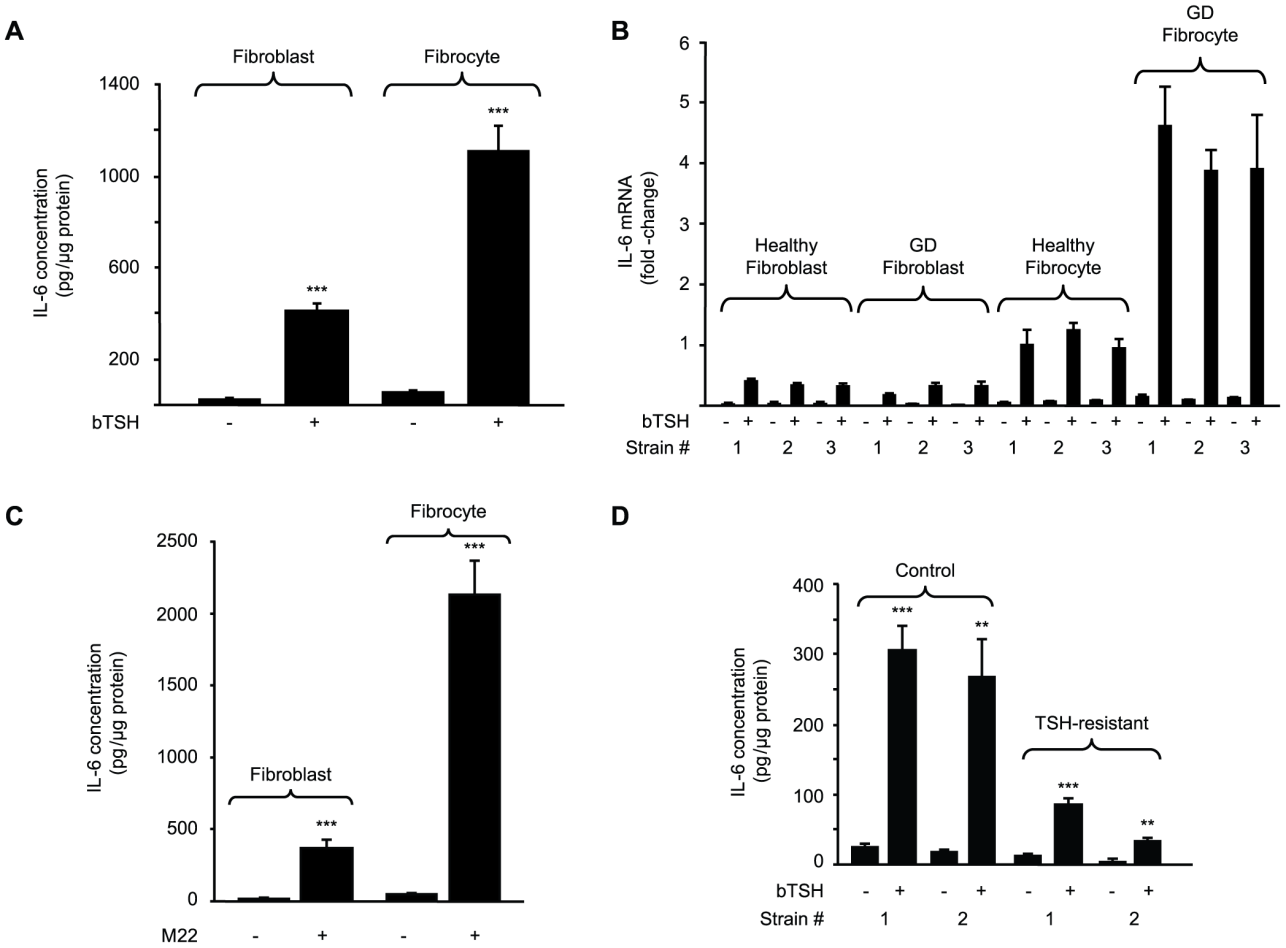
bTSH induces IL-6 in orbital fibroblasts and fibrocytes. (A) Confluent cultures were shifted to medium containing 1% FBS for 20 h and then treated without or with bTSH (5 mIU/mL) for 16 h. Media were collected and subjected to IL-6-specific ELISA. Cell layers were analyzed for protein content. Data are expressed as mean ± SD of three independent determinations (***, p<0.001). In a total of 3 experiments, IL-6 induction by bTSH was 18.1±5.1-fold in fibroblasts and 24.1±4.4-fold in fibrocytes. (B) Cultures were treated without or with bTSH for 6 h. Cellular RNA from three strains each of TAO orbital fibroblasts, healthy orbital fibroblasts, fibrocytes from healthy donors, and those with GD. Real-time RT-PCR was performed using the comparative critical threshold (C_T_) method. Ct values were normalized to respective GAPDH levels. Data are expressed as the mean±SD of three independent determinations. (C) IL-6 levels were determined as in panel A following treatment without or with M22 (2 µg/ml) for 16 h. (***, p<0.001). The induction of IL-6 by M22 in 3 experiments was 22.4±9.2-fold in fibroblasts and 47.1±10.1-fold in fibrocytes. (D) IL-6 levels were determined in fibroblasts from two individuals harboring a loss of function TSHR mutation (TSH-resistant) and two unaffected family members (control), treated without or with bTSH for 16 h as in panel A. Data are expressed as mean ± SD fold-change of three independent determinations (**, p<0.01; ***, p<0.001 vs untreated controls). In 3 separate experiments, TSH induced IL-6 by 12.7±1.6-fold and 14.5±1.6-fold in cultures from the healthy donors and 6.4±0.5 and 8.2±0.6-fold respectively in those from the two affected individuals.

Levels of basal IL-6 release from untreated orbital fibroblasts and fibrocytes is extremely low, as assessed by ELISA (Fig. 1A). bTSH (5 mU/ml) increased these levels in both cell-types after 16 h (23-fold, p<0.001) The induction is mediated at the pre-translational level (Fig. 1B). Steady-state IL-6 mRNA levels in three orbital fibroblast and fibrocyte strains each from healthy donors and those with GD are dramatically increased. The magnitude of these inductions was 14-fold (p<0.001), 20-fold (p<0.01), 16-fold (p<0.001), and 31-fold (p<0.001), respectively. Thus, responses in fibrocytes appear more vigorous than those in fibroblasts. Moreover, fibrocytes from patients with GD are considerably more responsive than those from healthy donors (p<0.001) (Fig. 1B).

**Figure 2:**
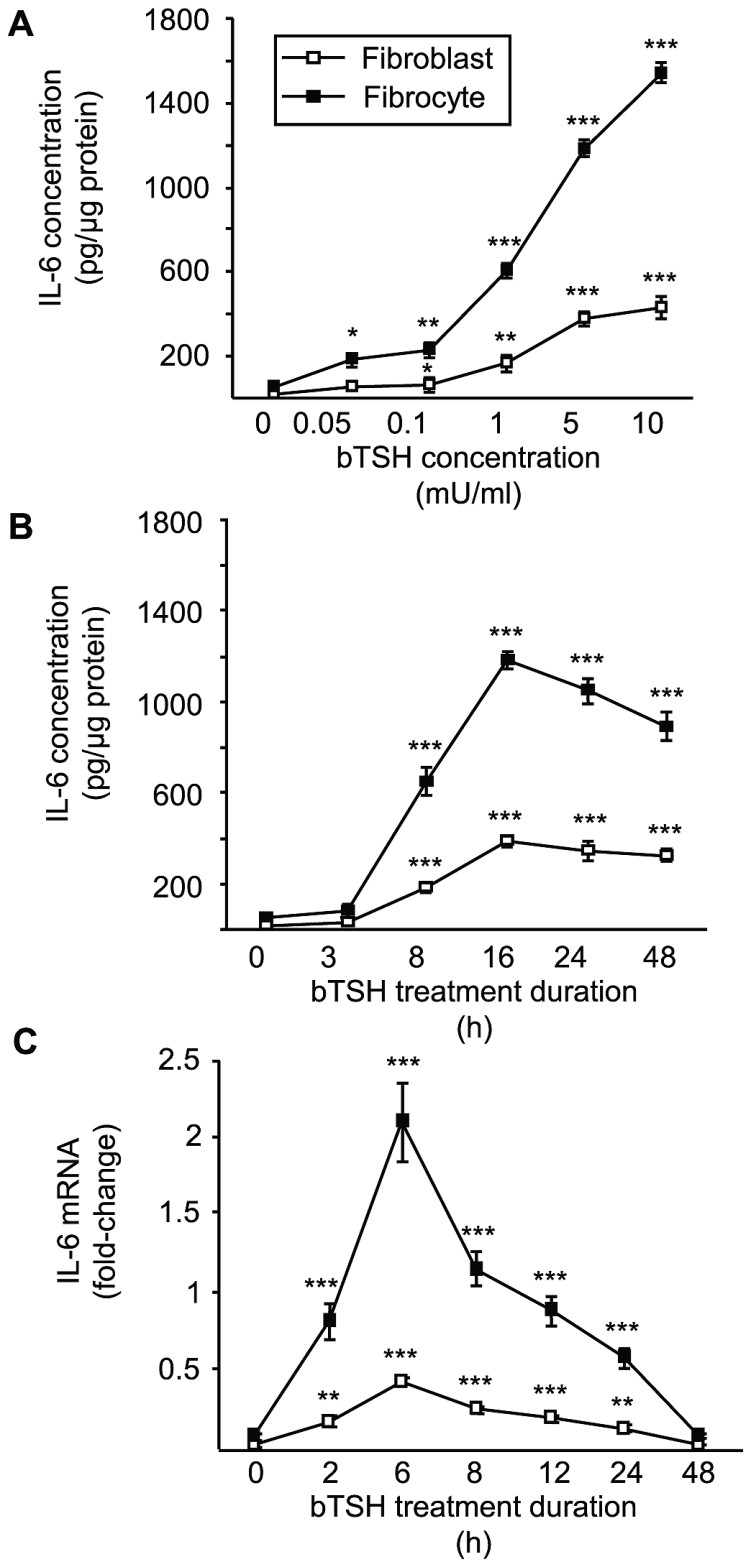
bTSH induction of IL-6 is concentration- and time-dependent. (A) Orbital fibroblasts and fibrocytes were treated with escalating concentrations of bTSH for 16 h. (B) Cells were treated with bTSH (5 mIU/mL) for graded intervals indicated along the abscissas. Media were collected and subjected to ELISA or (C) IL-6 mRNA levels determined by real-time RT-PCR. Data are expressed as mean ± SD of three independent determinations. (**, p<0.01; ***, p<0.001 vs baseline). Studies were performed three times.

Even at the lowest concentration of bTSH tested (0.05 mIU/mL), IL-6 levels are significantly increased in fibrocytes while the threshold of the effect was 0.1 mIU/ml in orbital fibroblasts (Fig. 2A). The response is near maximal at 5–10 mIU/mL where levels are more than 20-fold above untreated controls. These effects evolve rapidly and by 16 h, IL-6 is increased maximally (24-fold, p<0.0001) (Fig. 2B) while IL-6 mRNA peaks within 6 h (Fig. 2C). These levels return to baseline at 48 h.

**Figure 3:**
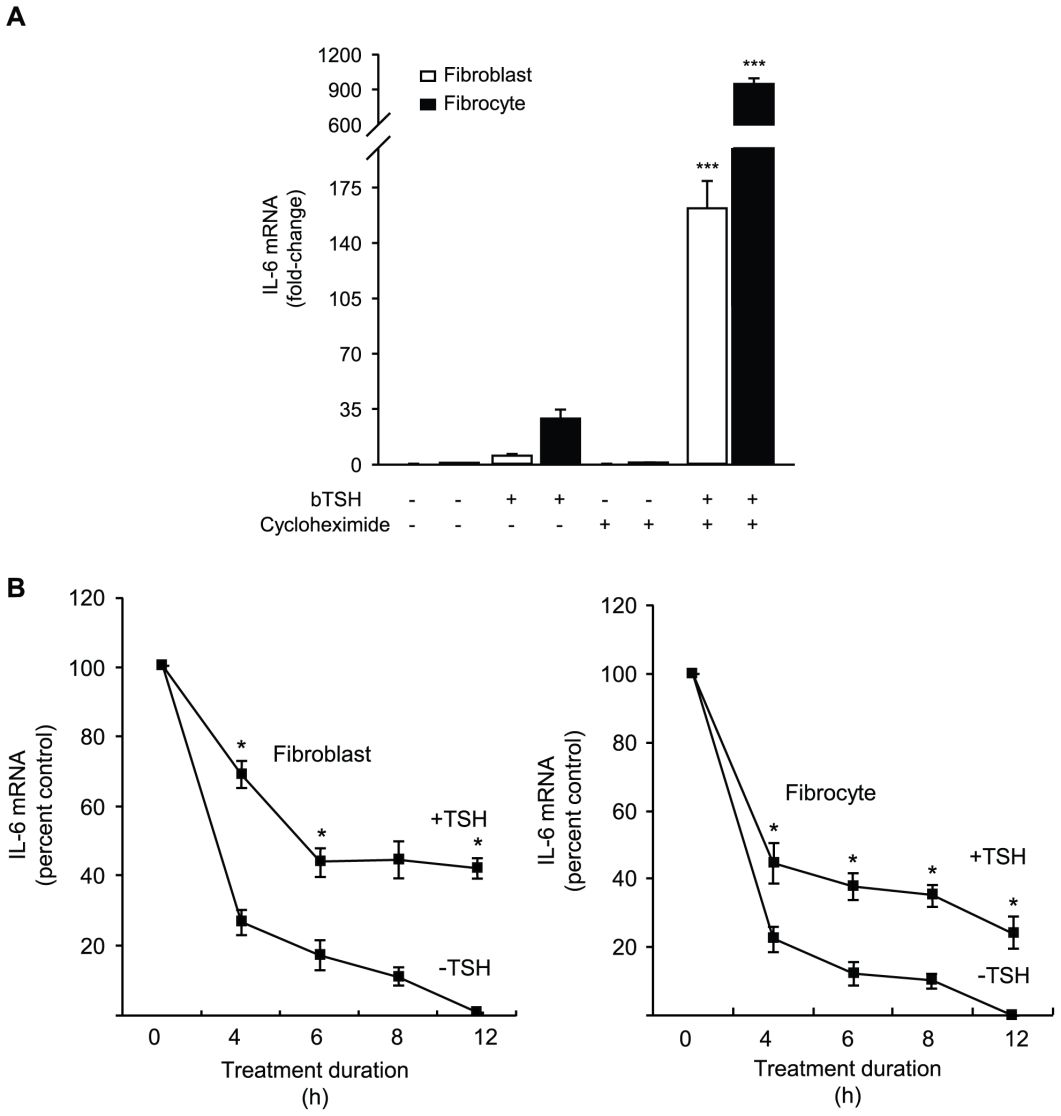
Effects of cycloheximide on the induction of IL-6 by TSH. Evidence that TSH delays IL-6 mRNA degradation. (A) Orbital fibroblasts and fibrocytes were treated with nothing or bTSH (5 mIU/mL) in the absence or presence of cycloheximide (10 µg/mL) for 6 h. Cellular RNA was isolated, and RT-PCR performed for IL-6. Ct values were normalized to GAPDH. Data are expressed as mean ± SD of three independent determinations. In 3 separate experiments, IL-6 production increased 31.8±2.8-fold in fibroblasts. **(**B**)** (left panel) Orbital fibroblasts and (right panel) fibrocytes were pretreated with bTSH (mIU/ml) for 12 h. Some culture wells were shifted to medium without TSH while the others were continued in its presence. All cultures received DRB (50 µM) at time “0” and were harvested at the times indicated along the abscissas. RT-PCR was performed and data graphed as a best fit line. Data are expressed as percent of transcript levels at time “0” ± SD of triplicate independent determinations, each from a separate experiment. (*, P<0.05 TSH-treated vs untreated cultures).

To determine whether the induction by TSH of IL-6 requires intermediate protein synthesis, cultures were treated without or with cycloheximide (10 µg/ml) in the absence or presence of bTSH. In fibroblasts, IL-6 mRNA is increased by 29-fold after a 6 h treatment with TSH (Fig. 3A). Cycloheximide fails to affect IL-6 mRNA levels when added as a single agent but when it is combined with TSH it results in a greater than 900-fold induction (p<0.0001 vs control). Results in fibrocytes are almost identical (p<0.0001). Thus, the induction of IL-6 by TSH represents a primary gene induction. Moreover, inhibiting protein synthesis results in a super-induction of IL-6, consistent with the presence of a constitutively active repressor protein in these cells.

Because TSIs rather than TSH activate TSHR in GD and drive hyperthyroidism [12], [13], these mAbs (M22, 2 µg/mL) were tested and found to induce IL-6 after 16 h by 22- and 46–fold in fibroblasts and fibrocytes, respectively (Fig. 1C) (both p<0.001). If the actions of TSH on fibroblasts are mediated through TSHR, fibroblasts from individuals with loss of function TSHR gene mutations may exhibit defects in their responses to TSH. Fibroblast strains from two affected and two unaffected members of a family with such a TSHR mutation [39] were incubated without or with bTSH for 16 h. As Fig. 1D demonstrates, TSH induces IL-6 6.5-fold (p<0.001 vs control) in the fibroblasts from those harboring the mutation while increasing levels 14.4-fold (p<0.001) in cells from the healthy family members (mutant vs healthy p = 0.013). Thus, it would appear that the induction of IL-6 in extra-thyroidal cells such as fibrocytes and orbital fibroblasts is mediated through TSHR and may carry substantial clinical relevance.

### TSH Stabilizes IL-6 mRNA

The influence of TSH on IL-6 mRNA stability was determined using DRB, an inhibitor of gene transcription [44]. bTSH retards IL-6 mRNA decay in both fibroblasts and fibrocytes (Fig. 3B). After 12 h, the duration of the study, approximately 50% of the transcript remains detectable in cultures treated with TSH while it becomes undetectable in its absence.

### G Protein Abundance, Adenylate Cyclase Expression, and cAMP Generation in Response to bTSH

**Figure 4:**
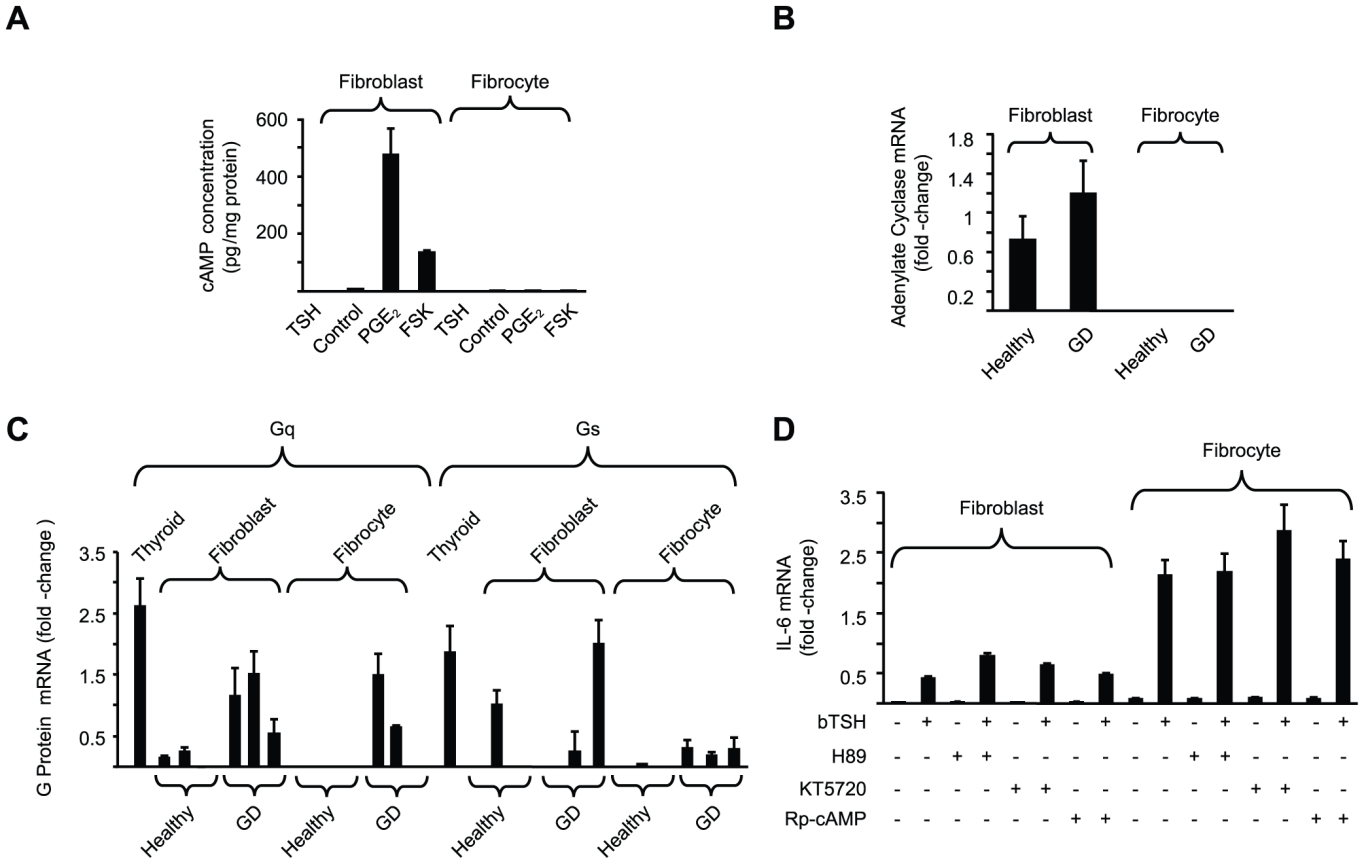
G protein, adenylate cyclase, and cAMP generation in orbital fibroblasts and fibrocytes. (A) Confluent cultures were treated with bTSH (5 mIU/mL), PGE_2_ (1 µM), forskolin (20 µM) or nothing (control) for 16 h. Cell layers were analyzed for cAMP content and protein determination. (B) Adenylate cyclase mRNA levels were determined in orbital fibroblasts and fibrocytes from healthy donors (n = 5) and those with GD (n = 5). (C) RNA from orbital fibroblasts, fibrocytes, and thyroid tissue was subjected to RT-PCR for Gq and Gs using the C_T_ method. Data are expressed as mean±SD of triplicate determinations from three different strains of each. (D) Cultures indicated were treated with nothing, bTSH, PKA inhibitors H89 (10 µM), KT5720 (10 µM) or Rp-cAMP (1 mM) alone or in the combinations indicated for 6 h. RNA was extracted and subjected to RT-PCR for IL-6. Signals were normalized to GAPDH. Data are expressed as the mean ± SD of fold-change in three independent determinations from a single experiment, representative of three experiments performed. In 3 separate experiments, H89 failed to inhibit TSH-dependent IL-6 expression (1.62±0.23-fold and 1.06±0.3-fold increase in fibroblasts and fibrocytes, respectively vs TSH alone).

The dominant signaling downstream from TSHR in thyroid cells involves generation of cAMP [45]. To ascertain whether the same is true in other cells, fibroblasts and fibrocytes were treated with bTSH (5 mU/ml) and cAMP generation determined (Fig. 4A). TSH elicits low amplitude cAMP generation in fibroblasts, a response that was completely absent in fibrocytes. Prostaglandin E_2_ (PGE_2_) induces IL-6 through a cAMP-dependent mechanism in orbital fibroblasts [46]. PGE_2_ and forskolin increase cAMP levels in these fibroblasts but neither agent provokes detectable cAMP in fibrocytes. Adenylate cyclase mRNA is detected in orbital fibroblasts from both healthy donors and those with GD (Fig. 4B). In stark contrast, the transcript is completely undetectable in 10 different fibrocyte strains, regardless of donor health status (Fig. 4B). Thus, the complete absence of cAMP generation in response to any of the agents tested is explained by the fibrocytes lacking adenylate cyclase expression.

Expression of Gq and Gs mRNAs were next assessed since TSHR couples to both in thyroid cells [45]. Gq and Gs are variably expressed in the fibroblast and fibrocyte strains examined. As Fig. 4C indicates, Gq transcripts are detected in a majority of orbital fibroblast strains and in two of three GD fibrocyte strains but are undetectable in healthy fibrocytes. Expression of Gs mRNA is sporadic in both cell types and absent or at an extremely low level in fibrocytes. As expected, transcripts encoding Gq and Gs are abundant in thyroid tissue. Rp-cAMP, a specific cAMP antagonist and competitive PKA inhibitor [47] fails to alter TSH-induced IL-6 mRNA in either fibroblasts (p = 0.69) or fibrocytes (p = 0.77) (Fig. 4D). Similarly, PKA inhibitors H89 and KT5720 have no effect on TSH-dependent IL-6 expression. These inhibitors were previously shown to be active in TAO fibroblasts treated with PGE_2_ [46]. Thus, cAMP production and the PKA pathway do not appear to participate in IL-6 induction by TSH in either cell type.

### bTSH-induced IL-6 production is Mediated Through AKT and PKC Pathways

**Figure 5:**
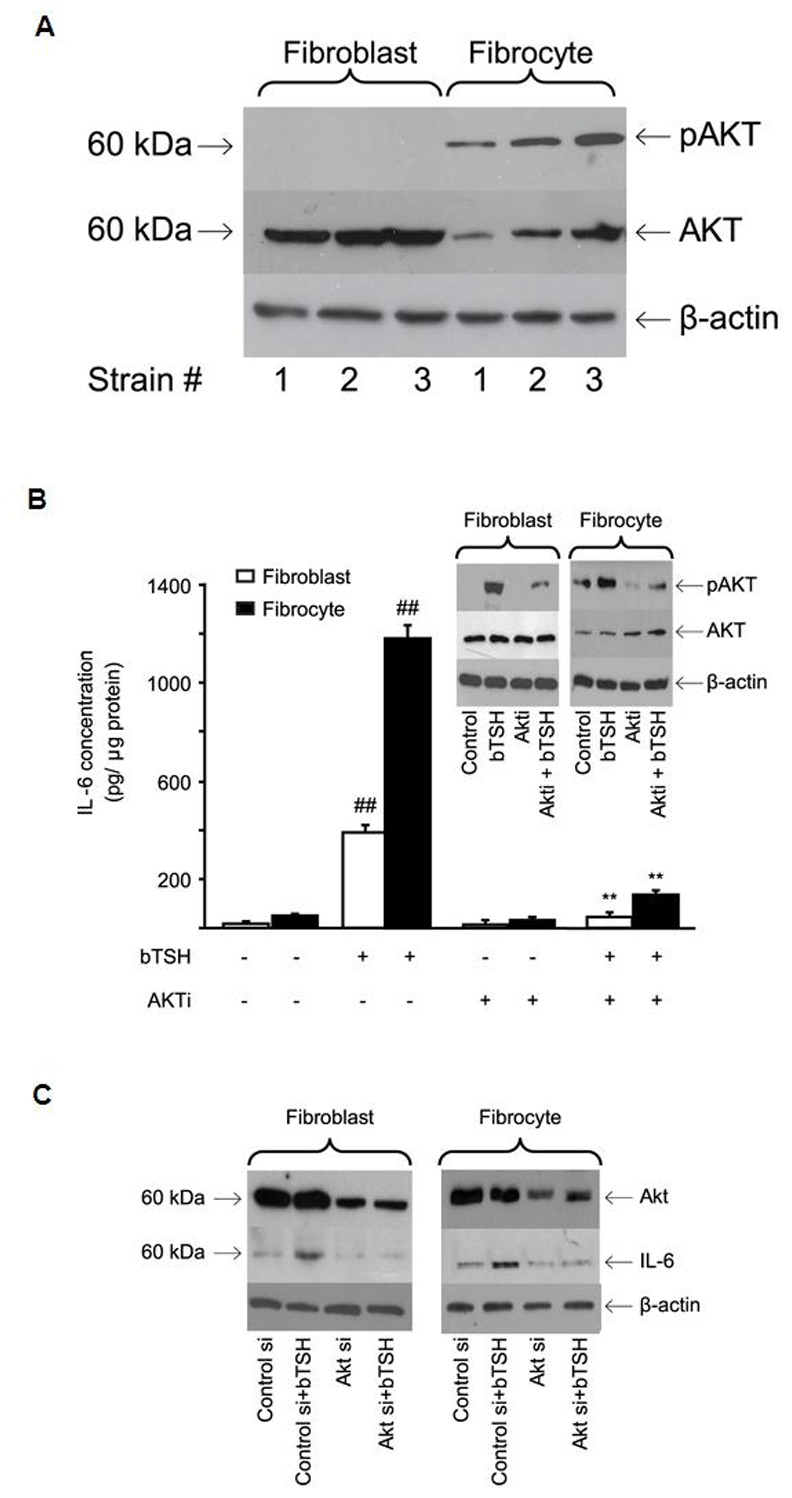
Role of AKT in the induction by TSH of IL-6. (A) Cellular proteins harvested from untreated cultures from 3 different donors each were subjected to Western blot analysis for AKT and pAKT Ser 473 as described in Experimental Procedures. (B) Confluent orbital fibroblast cultures, in this case from a patient with TAO, and fibrocytes from a healthy donor, were treated without or with bTSH (5 mIU/mL) in the absence or presence of AKTi (1 µM) for 16 hrs. Media were analyzed for IL-6 and cell layers for protein content. Data are presented as the mean ± SD of three independent determinations. (##, P<0.01 compared to untreated cultures; **, P<0.01 compared to TSH-treated cultures). In 3 separate experiments, Akti inhibited TSH-dependent IL-6 expression by 83±5% and 84±4% in fibroblasts and fibrocytes respectively. (Inset) TSH provokes AKT Ser 473 phosphorylation that is inhibited by AKTi. Cultures were treated with nothing or the agents indicated for 30 min. and cell protein was subjected to Western blotting. Densitometric analysis for pAKT bands: bTSH-treated fibroblasts, 29±3 AU; bTSH+AKTi, 9±2 AU. Fibrocytes, 34±5.5 AU and 14±2.7 AU, respectively. In 3 separate experiments, Akti inhibited pAKT by 67±7% and 58±9% in fibroblasts and fibrocytes, respectively. (C) knockdown of AKT with specific siRNA attenuates bTSH-induced IL-6. siRNA targeting AKT or control (scrambled) siRNA was transfected into 80% confluent cultures. After 48 h, these were treated without or with bTSH (5 mIU/mL) for 16 h. Protein was subjected to Western blot analysis. In 3 separate experiments, AKT siRNA reduced TSH-dependent IL-6 by 53±4% and 49±3% in fibroblasts and fibrocytes, respectively.

AKT plays an important role in mediating TSH actions in thyroid epithelial cells [48]. This pathway was therefore examined in fibroblasts and fibrocytes. AKT levels are considerably higher in fibroblasts than fibrocytes (Fig. 5A). In contrast, fibrocytes exhibit basal phosphorylation of AKT whereas fibroblasts do not. AKTi (1 µM) blocks TSH induction of IL-6 (Fig. 5B). In addition, it attenuates the TSH-dependent phosphorylation of AKT at Serine 473 in both cells (Fig. 5B, Inset). Like AKTi, AKT-targeting siRNA attenuates IL-6 induction by bTSH (Fig. 5C).

**Figure 6:**
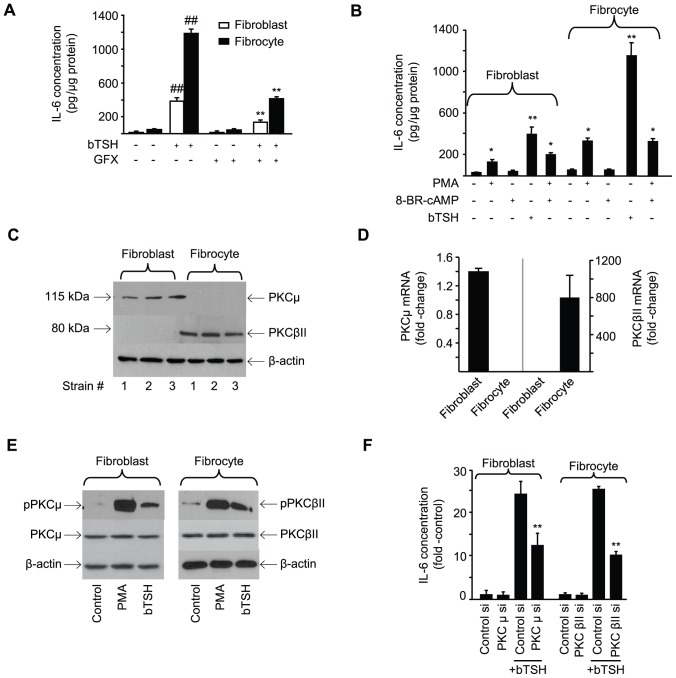
Role of PKC in the induction by TSH of IL-6. (A) Confluent orbital fibroblasts and fibrocytes were treated with TSH alone or in combination with GFX (10 µM) for 16 h. Media were subjected to an IL-6 ELISA. Data are expressed as mean ± SD of triplicate independent determinations. (##, P<0.01 vs untreated controls **, P<0.01 vs TSH alone). In 3 separate experiments, GFX inhibited TSH-provoked IL-6 expression by 64.2±5.1% and 65.9±4.8% in fibroblasts and fibrocytes, respectively. (B) Cultures were treated with nothing, bTSH, 8-Br-cAMP (1 mM), PMA (50 ng/mL) or the combination indicated for 16 h. Media were analyzed for IL-6 content (**, P<0.01 vs untreated controls). (C). Cellular protein from three strains of each cell type were subjected to Western blot analysis of PKCµ and PKCβII. (D) RNA was extracted from the cell types indicated and subjected toRT-PCR for PKCµ and PKCβII mRNA by the C_T_ method. Signals were normalized to GAPDH. Data are expressed as the mean ± SD of fold-change in three independent determinations from a single experiment, representative of three experiments performed. (E) Cultures were treated with nothing, bTSH (5 mIU/mL), or PMA (50 ng/mL) for 30 min, harvested, and proteins analyzed by Western blot for PKCµ, pPKCµ (Ser 916), PKCβII, and pPKCβII (Ser 660). Results are from a single experiment, representative of three performed. (F) Targeting siRNAs and their scrambled counterparts were transfected into sub- confluent monolayers as described in Experimental Procedures. After 48 h, they were treated with nothing or bTSH (5 mIU/mL) for 16 h. Media were collected and subjected to an IL-6 ELISA. Data are expressed as the mean ± SD of three independent determinations from a single experiment, representative of three performed. **, p<0.01 vs TSH-treated cultures transfected with control siRNA. In 3 separate experiments, PKCµ siRNA inhibited TSH-provoked IL-6 by 48.5±1% in fibroblasts and PKCβII siRNA inhibited TSH-induced IL-6 by 59± % in fibrocytes.

Several factors known to induce IL-6 expression act through PKC [49], [50]. GFX, a pan-PKC inhibitor [51], attenuates IL-6 induction by bTSH (64% inhibition in both cell types, p<0.01) (Fig. 6A). On the other hand, phorbol 12-myristate 13-acetate (PMA) (100 ng/mL) could mimic TSH effects on IL-6, although the magnitude is considerably less (Fig. 6B). This disparity suggests that additional signaling pathways besides PKC may be necessary for maximal induction of IL-6 by TSH. 8-Br-cAMP added alone or in combination with PMA, fails to influence IL-6 in fibrocytes.

The PKC isoenzymes relevant to IL-6 induction by bTSH were next identified. PKCµ is easily detected in orbital fibroblasts but appears absent in fibrocytes (Fig. 6C). In contrast, PKCβII is expressed abundantly by fibrocytes but not by fibroblasts. The divergence of PKC isoenzyme expression is the consequence of dramatically different mRNA levels in fibroblasts and fibrocytes (Fig. 6D). The respective enzymes mediate the induction by TSH of IL-6 in a cell-specific manner. First, phosphorylated PKCµ Ser 916 and PKCβII Ser 660 can be detected following bTSH treatment in fibroblasts and fibrocytes, respectively (Fig. 6E). siRNA targeting PKCµ attenuates IL-6 production in orbital fibroblasts while interrupting PKCβII blocks the induction of IL-6 in fibrocytes (Fig. 6F).

### PDK1 Plays a Critical Role in TSHR Signaling in Orbital Fibroblasts and Fibrocytes

**Figure 7:**
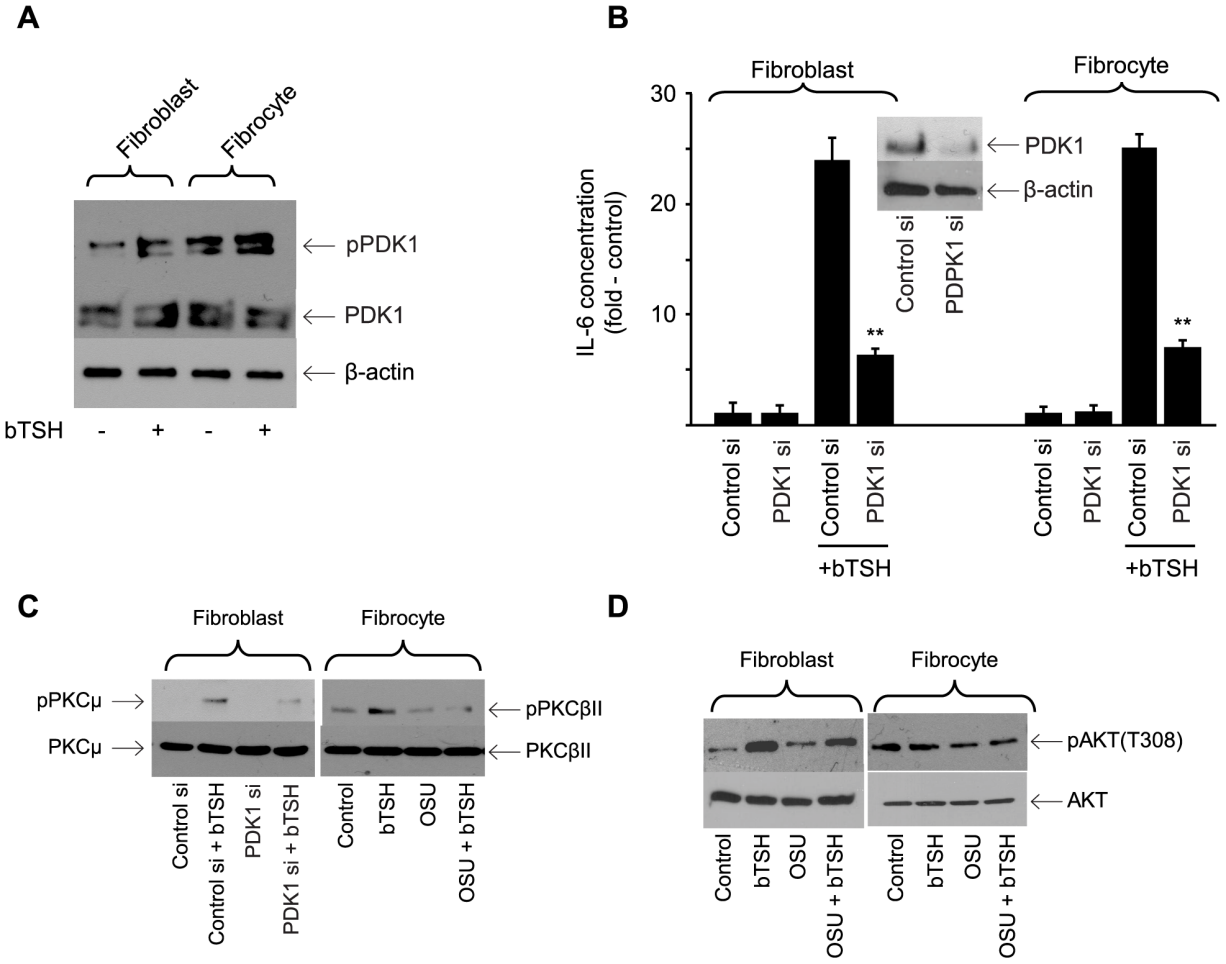
Involvement of PDK1 in the induction by TSH of IL-6. (A) Cultures were untreated (control) or bTSH (5 mIU/ml) was added to medium for 30 min. Cellular protein was subjected to Western blot analysis for PDK1 and pPDK1 and re-probed for β-actin. Results are representative of three separate experiments performed. Densitometric analysis; pPDK1, control fibroblasts, 16.25±2.85 AU; plus bTSH, 42.7±6.54 AU; control fibrocytes, 64.5±2.67 AU; plus TSH, 78.5±3.8 AU. In 3 separate experiments, TSH increased pPDK1 levels by 2.6±0.3-fold and 1.2±0.3-fold in fibroblasts and fibrocytes, respectively. (B) Sub- confluent cultures were transfected with either control siRNA or one targeting PDK1, followed by 48 h incubation. Cultures were treated with nothing or bTSH (5 mIU/mL) for 16 h, media collected and subjected to IL-6-specific ELISA and cell layers analyzed for protein content. Data are expressed as the mean ± SD of three independent determinations. Inset: Cell layers were subjected to Western blot analysis for PDK1 after transfection with control siRNA or that targeting PDK1. In 3 separate experiments, PDK1 siRNA reduced TSH-induced IL-6 levels by 73±4% in fibroblasts and 73±5% in fibrocytes. (C) Orbital fibroblasts, in this case from a patient with TAO, were transfected with PDK1siRNA while fibrocytes were treated with OSU-03012 (5 µM) for 6 h. Cultures were treated as indicated (bTSH, 5 mIU/mL) for 30 min. Cellular protein was subjected to Western blot analysis of PKCµ and pPKCµ in fibroblasts (left panel) and PKCβII and pPKCβII in fibrocytes (right panel). (D) Confluent cultures were pre-treated without or with OSU-03012 (5 µM) for 6 h, then treated with nothing (control) or bTSH (5 mIU/ml) for 30 min. Cellular proteins were subjected to Western blot analysis probing with AKT and pAKT antibodies. Inhibition of TSH-dependent pAKT by OSU-03012 in 3 separate experiments was 14.4±1.2% and 2.5±0.6% in fibroblasts and fibrocytes, respectively.

PDK1 activates AKT and PKC in several cell-types [52], [53]. Basal phosphorylation of the protein is considerably greater in fibrocytes than in orbital fibroblasts (Fig. 7A). bTSH (5 mU/ml) provokes phosphorylation of PDK1 in both cell types. siRNA targeting PDK1 attenuated IL-6 production by 74% (p<0.001 vs control siRNA) in both cells (Fig. 7B). The siRNA targeting PDK1 attenuates TSH-dependent PKCµ Ser 916 phosphorylation in fibroblasts (Fig. 7C) while the PDK1 inhibitor, OSU-03012 (5 µM) [54] blocks TSH-dependent phosphorylation of PKCµ Ser 916 and PKCβII Ser 660 levels in the respective cell-types (Fig. 7C). The inhibitor also reduces levels of TSH-dependent pAKT Threonine 308 in fibroblasts but fails to do so in fibrocytes (Fig. 7D, 14.4±1.2% and 2.5±0.6%, respectively). Thus it would appear that PDK1 represents an important kinase in TSHR signaling upstream from PKC in both cell types. Its involvement in basal and TSH-dependent phosphorylation of AKT at Threonine 308 appears to diverge in the two cell-types.

### Transition from PKCβII Expression in Fibrocytes to PKCµ in Orbital Fibroblasts may be Imposed by CD34^−^ Fibroblasts

**Figure 8:**
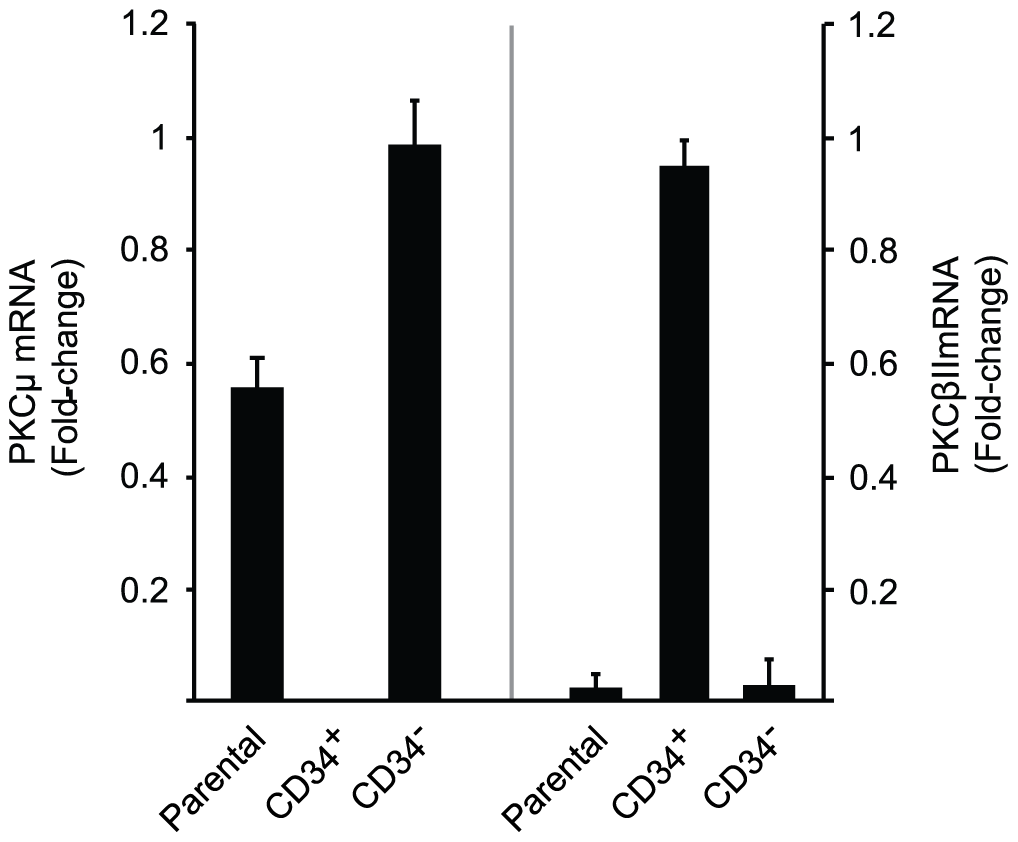
Divergent PKCµ and PKCβII mRNA expression in pure CD34^+^ and CD34^−^ orbital fibroblast subsets. A parental strain of TAO orbital fibroblasts (containing mixed CD34^+^ and CD34^−^ cells) was sorted into pure CD34^+^ and CD34^−^ subsets by FACS as described in Experimental Procedures. These were then cultured for 48 h., RNA was isolated, andRT-PCR performed for (left panel) PKCµ and (right panel) PKCβII using the C_T_ method. Ct values were normalized to their respective GAPDH levels. Data are expressed as the mean ± SD of three independent replicates from a single experiment, representative of three performed. PKCµ mRNA was undetectable in CD34^+^ subsets in all 3 studies while PKCβII mRNA levels were 44±7-fold above their respective parental fibroblast cultures.

CD34^+^ orbital fibroblasts appear to derive from circulating CD34^+^ fibrocytes [34]. Moreover, as fibrocytes transition to CD34^+^ fibroblasts, their phenotype appears to be modified as a consequence of interactions with CD34^−^ fibroblasts [36]. We thus examined whether the pattern of PKC isoenzyme expression in CD34^+^ fibroblasts (PKCµ) might revert to that of fibrocytes (PKCβII) following separation into a pure CD34^+^ subset. A parental strain of TAO orbital fibroblasts (mixed CD34^+^ and CD34^−^) was sorted into pure CD34^+^ and CD34^−^ subsets. The mixed strains express PKCµ mRNA, as anticipated, and PKCβII mRNA remains undetectable (Fig. 8). This pattern of PKCµ expression is also found in CD34^−^ cells. In contrast, pure CD34^+^ fibroblasts express PKCβII while PKCµ mRNA becomes undetectable. Thus, it would appear that removing CD34^−^ fibroblasts restores the pattern PKC expression in CD34^+^ fibroblasts to one resembling that of fibrocytes.

### Up-regulation of IL-6 Expression by bTSH Involves Activation of the IL-6 Gene Promoter

**Figure 9:**
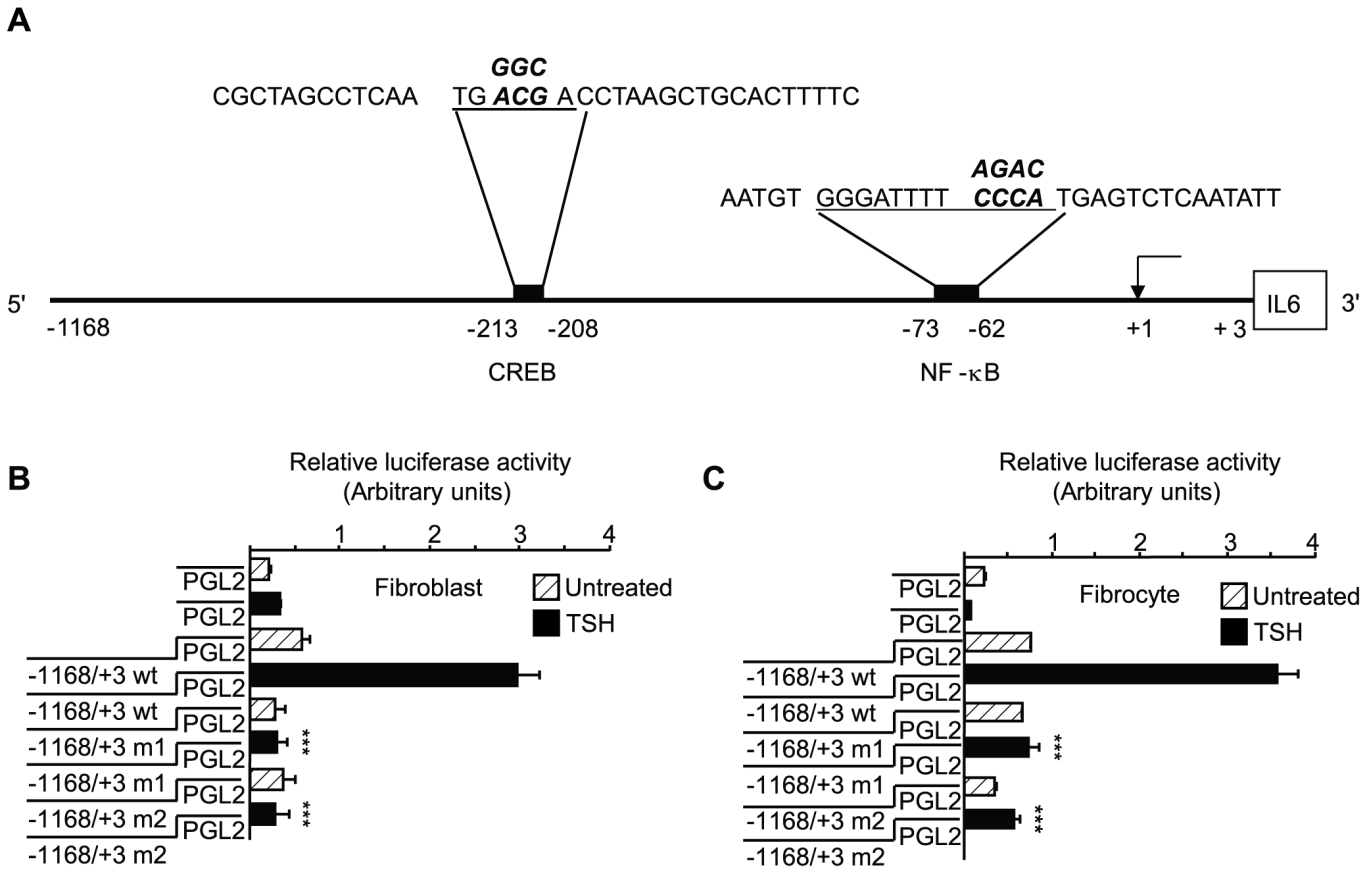
CREB and NF-κB sites are critical to activation of the IL-6 gene promoter by bTSH. (A). Schematic demonstrating *cis*-acting regulatory elements for CREB (TGACGA, −213 to −208 nt) and NF-κB (GGGATTTTCCCA, −73 to −62 nt) relative to the transcription start site (+1). These are underlined and base substitutions resulting from site-directed mutagenesis appear above those in the corresponding wild-type sequences (emboldened). (B) Orbital fibroblasts and (C) fibrocytes were transiently transfected with empty luciferase vector or one containing the 1171 nt fragment spanning −1168 to +3 nt of the human IL-6 gene promoter, or that fragment harboring a 3 base mutation in the CREB binding site (designated “m1”), or a 4 base mutation in the NF-κB binding site (designated “m2”). Sub-confluent cultures were then treated with nothing (control) or bTSH (5 mIU/mL) for 1 h., cell layers harvested and luciferase reporter activity assessed in a luminometer. Data are expressed as the mean ± SD of triplicate determinations from a single experiment, representative of three performed. ***, P<0.001 versus TSH-treated cells transfected with wt promoter fragment. In 3 separate experiments, the m1 mutation resulted in an 86±4% and 79±4% reduction in TSH-dependent promoter activity in fibroblasts and fibrocytes respectively compared to wild type. The m2 mutation resulted in an 87±4% and 84±4% reduction, respectively.

To determine whether TSH upregulates IL-6 gene transcription, a fragment of its promoter, extending from −1168 nt to +3 nt (Fig. 9A), was cloned, fused to a luciferase reporter gene, and transiently transfected into fibrocytes and fibroblasts. bTSH increases promoter activity at 1 h by 5-fold and 4.7- fold, respectively, compared to untreated controls (Figs. 9B and 9C, p<0.001 and p<0.01). Because many TSH actions are mediated through CREB as well as NF-κB [55]–[58], the single CREB binding site identified in this promoter fragment, extending from −213 to −208 nt, was mutated (Fig. 9A). Mutating *ACG* to *GGC* and yielding the promoter fragment designated “m1” dramatically attenuates TSH-dependent promoter activity in fibroblasts and fibrocytes by 86% and 79%, respectively, (p<0.001 vs wild-type) (Figs. 9B and 9C). Next, the NF-κB site extending (−73 to −62 nt) [58] was mutated from *CCCA* to *AGAC* (fragment “m2”). This mutation too substantially reduces activity (87% and 84%, p<0.001 vs wt, respectively). Thus it would appear that both CREB and NF-κB are both essential to the induction of IL-6 by TSH.

**Figure 10:**
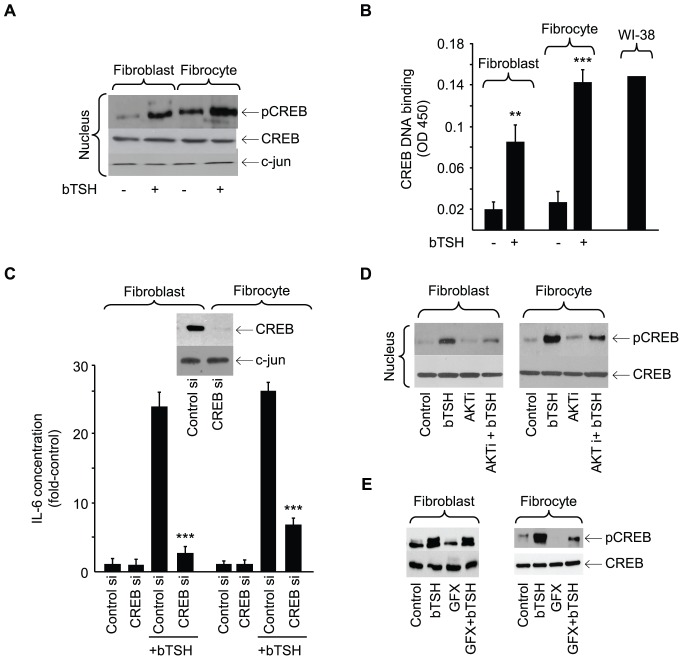
Involvement of CREB in the induction of IL-6 by bTSH. (A) Orbital fibroblasts and fibrocytes were treated without or with bTSH for 15 min, nuclear protein was extracted and probed for CREB, pCREB, and c-jun by Western blot. (B) Sub-confluent orbital fibroblast and fibrocyte cultures were treated without or with bTSH for 16 h. Nuclear extracts (5 µg) were subjected to the TransAM ELISA for CREB/DNA complex abundance, as determined at 450 nm. Data are expressed as the mean ± SD of triplicate independent determinations from single fibroblast and fibrocyte strains. Three separate studies yielded the following: fibroblasts, TSH 4.4±0.8-fold vs control (p<0.01); fibrocytes, TSH 5.4±0.6-fold vs control (p<0.001). Forskolin-treated WI-38 cells served as positive control. (C) Sub-confluent cultures were transfected with either control siRNA or one targeting CREB, followed by 48 h incubation. Cultures were treated with nothing or bTSH (5 mIU/mL) for 16 h, media collected and subjected to IL-6-specific ELISA. Inset: Cell layers from cultures transfected with control and CREB-targeting siRNAs were subjected to Western blot analysis for CREB. Data are expressed as the mean ± SD of three independent determinations. In 3 separate experiments, CREB siRNA inhibited TSH-induced IL-6 by 2.3±0.5-fold and 2.5±0.6-fold in fibroblasts and fibrocytes, respectively. (D) Cultures were pre-treated without or with AKTi (1 µm) for 1 h, then treated with nothing or bTSH (5 mIU/mL) for 30 min. Cellular proteins were subjected to Western blot analysis for CREB and pCREB. Results are from one study, representative of three performed. Densitometric analysis of pCREB/CREB bands: bTSH treated fibroblasts, 0.947±0.073 AU vs plus AKTi, 0.59±0.09 AU. bTSH treated fibrocytes, 2.72±0.33 AU vs plus AKTi, 1.31±0.38 AU. In 3 separate experiments, AKTi reduced levels of TSH-induced pCREB by 66±7% and 58.4±8.7% in fibroblasts and fibrocytes, respectively. (E). Cultures of orbital fibroblasts, in this case from a patient with TAO, (left Panel) and fibrocytes (right panel) were treated with nothing or bTSH (5 mIU/mL) in the absence or presence of GFX (10 µM) for 30 min. Nuclear protein fractions were probed for pCREB and CREB by Western blotting. Densitometric analysis of pCREB/CREB bands revealed: bTSH treated fibroblasts, 1.02±0.08 AU vs bTSH plus GFX, 0.82±0.04 AU. Fibrocytes, 1.36±0.21 AU vs 0.75±0.23 AU, respectively. In 3 separate experiments, GFX inhibited TSH-provoked pCREB levels by 18±3% and 48±7% in fibroblasts and fibrocytes, respectively.

**Figure 11:**
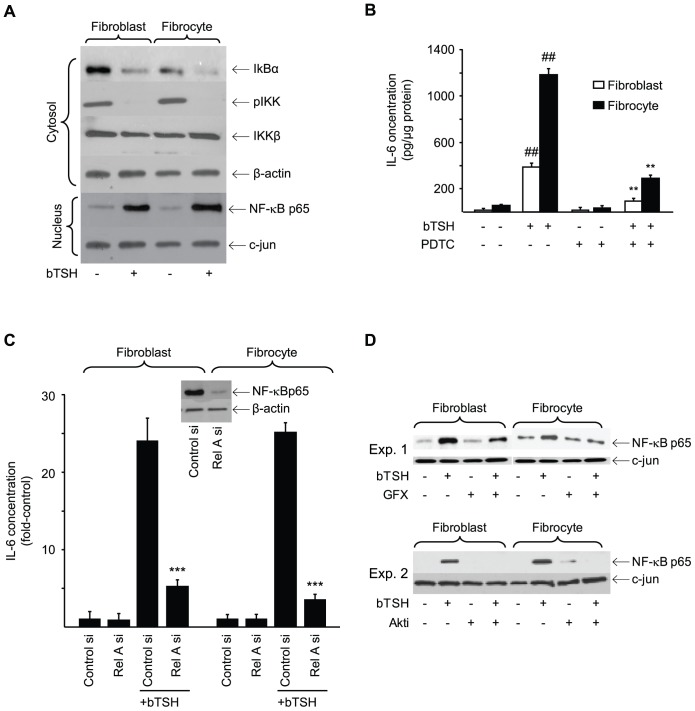
Involvement of NF-κB in the induction of IL-6 by bTSH. (A) Confluent orbital fibroblast and fibrocyte cultures were treated without or with bTSH for 60 min. Cytosolic and nuclear protein fractions were prepared as described in Experimental Procedures. Nuclear protein extract was probed with anti-NF-κB-p65 Abs by Western blot analysis. Densitometry: nuclear p65, control vs bTSH-treated fibroblasts, 11.72 AU vs 58.5 AU; fibrocytes, 10.2 AU vs 75.3 AU, respectively. Cytosolic extracts were subjected to Western blot analysis of IκBα, pIKK, and IKKβ. (B) Orbital fibroblasts, in this case from a patient with TAO, and fibrocytes were treated with nothing or bTSH (5 mIU/mL) in the absence or presence of PDTC (100 µM) for 16 h. Media were analyzed for IL-6 content by ELISA. Data are presented as the mean ± SD of 3 independent determinations. (##, P<0.01 vs untreated controls; **, P<0.01 vs TSH alone). (C) Sub-confluent orbital fibroblast and fibrocyte cultures were transfected with control siRNA or that targeting Rel A, incubated for 48 h. and then treated with nothing or bTSH (5 mIU/mL) for 16 h. Media were analyzed for IL-6. Inset: Western blot confirming knockdown of Rel A. Data are expressed as the mean ± SD of three independent determinations. ***, p<0.001 vs control siRNA. (D) Orbital fibroblasts and fibrocytes were treated with nothing or bTSH (5 mIU/mL) in the absence or presence of GFX (10 µM) or Akti (1 µM) for 30 min. Nuclear protein fractions were prepared as described in Experimental Procedures and probed with anti-NF-κB-p65 Abs by Western blotting. Densitometry: Fibroblasts bTSH vs bTSH plus GFX, 0.826±0.196 AU vs 0.485±0.06 AU, bTSH vs bTSH plus AKTi, 0.253±0.02 AU vs 0.0 AU; Fibrocytes, 0.575±0.072 AU vs 0.391±0.056 AU and 0.485±0.03 AU vs 0.043±0.012 AU respectively. In 3 separate experiments, GFX reduced TSH-dependent p65 levels by 42±7% and 32±8% in fibroblasts and fibrocytes, respectively. AKTi reduced these levels by 91±1% in fibrocytes.

bTSH treatment for 15 min increases nuclear pCREB content (Fig. 10A). Congruently, DNA binding activity exhibited by CREB is also increased (Fig. 10B, fibroblasts, 4.3-fold, p<0.01; fibrocytes, 5.4-fold, p<0.001). Moreover, siRNA targeting CREB attenuates the induction of IL-6 by 56% (p<0.001) and 61%, respectively (p<0.001) (Fig. 10C). Activated AKT lies upstream from phosphorylated CREB [59]. AKTi attenuates TSH-provoked CREB phosphorylation in both cell-types (67% and 59%, respectively) (Fig. 10D). In contrast, GFX reduces TSH-dependent CREB phosphorylation in fibrocytes but failed to do so in fibroblasts (Fig. 10E). TSH also provokes nuclear translocation of p65 and reduces the cytosolic content of IκBα and pIKK (Fig. 11A). The NF-κB inhibitor, PDTC, inhibits IL-6 induction by 75% (p<0.01) (Fig. 11B). Knocking down p65-RelA with a specific siRNA also attenuates the IL-6 induction in both cell types (p<0.001, Fig. 11C). GFX reduces nuclear p65 content in both fibroblasts and fibrocytes (Fig. 11D, Expt.1) while AKTi does the same (Fig. 11D, Expt.2).

## Discussion

Orthodoxy has taught that TSHR expression is limited to the thyroid epithelium [1]. This lesson began to change when reports appeared that the receptor could be detected, albeit at relatively low levels, in lymphocytes, skin, and several fatty connective tissue depots [4], [7]. TSH induces IL-6 release from cultured 3T3-L1 cells [26] and human adipocytes [27] but the mechanisms involved have not been explored in detail. Identification of high-level functional TSHR expression by fibrocytes provides renewed impetus for systematically exploring post-receptor signaling pathways utilized in extra-thyroidal cells [34]–[37]. TSHR levels in TAO orbital fibroblasts are considerably lower than those in fibrocytes [36], [60], yet bTSH induces substantial IL-6 production (Figs.1A and 1B) in both cell-types through an up-regulation of gene promoter activity and stabilization of IL-6 mRNA. Our current findings demonstrate that multiple signaling pathways are involved and provide evidence that cross talk between pathways plays an important role. They strongly suggest that the pattern of signaling may diverge from that described previously in thyroid epithelium where cAMP generation results in activation of c-Jun N-terminal kinase, AKT, ERK1/2 and Rap1 through the PKA regulatory subunit, RIIβ [61], [62]. In addition to those actions of TSH in thyroid epithelium requiring PKA activation, effects that appear to be completely independent of cAMP generation are emerging. In particular, those initiated by Gβγ are involved with thyroid differentiation and AKT activation in a phosphoinositide 3-kinase (PI3K)-dependent and cAMP independent manner [48]. Activation of this pathway reduces the expression of the sodium/iodine symporter through an impact on nuclear PAX-8 content. But PI3K can also be activated by Ras which in turn can be activated by cAMP [63]. Thus the complex cross-talk occurring between cAMP-dependent and -independent pathways can confound the interpretation of results in studies attempting to partition the contribution of specific kinase cascades. This is particularly relevant to dissecting aspects of TSHR signaling that are completely independent of cAMP generation. For this purpose, the fibrocyte appears to represent an outstanding cell model. Adenylate cyclase mRNA is undetectable in these cells and TSH, PGE_2_, and forskolin all fail to provoke detectable cAMP generation (Fig. 4A). Further, exposure of these cells to exogenous cAMP also fails to induce IL-6 in fibrocytes, reinforcing the conclusion that TSH must be inducing the cytokine through cAMP-independent pathways. The absence of cAMP-dependent pathways in fibrocytes thus allows a very clear delineation of TSH signaling that relies entirely on other cascades. Similarly, those molecular events that exhibit complex, dual regulation with respect to cAMP, such as the regulation of Rap1 by TSH [64], might be further interrogated in fibrocytes to further delineate involvement of multiple signaling pathways. The current findings are congruent with earlier reports by Saunier *et al* [65]. They found that activation of type II deiodinase in astroglial cells by TSH is mediated through cytosolic phospholipase A_2_ and that TSH activates ERK through pertussis toxin-insensitive and cAMP-independent mechanisms [66].

From these studies it is evident that AKT, PKC, and PDK1 play dominant roles in post-receptor TSHR signaling in fibroblasts and fibrocytes (Figs. 5–7). But differences emerge between the two cell-types. Basal levels of pAKT (T308 and Ser 473), pPKC, and pPDK1 are considerably higher in fibrocytes than in fibroblasts. This entire cAMP-independent pathway is thus considerably more active in fibrocytes. Another unanticipated divergence relates to the pattern of PKC isoenzyme expression and usage. Fibrocytes and their derivative pure CD34^+^ orbital fibroblasts express and utilize PKCβII. Interruption of this kinase attenuates TSH signaling (Fig. 6C–6F) while unsorted TAO orbital fibroblasts express PKCµ/PKD, a member of another serine/threonine kinase family [67]. These two distinct enzymes exhibit divergent structural and functional attributes, including substrate specificity [68] and inhibitor susceptibility. They possess dissimilar kinase pseudomains and PKCµ more closely resembles calmodulin-dependent protein kinase II protein kinases [69]. It appears that some factor(s) emanating from CD34^−^ fibroblasts promotes PKCµ expression/usage (Fig. 8). Switching from one enzyme to another may determine the functional phenotype of these fibroblasts. This is the case in models of cardiac hypertrophy [70] and cancer susceptibility [71], [72] where divergent PKC usage impacts cell-specific function. Inhibiting PKC with the pan-PKC-targeting agent, GFX, which also targets PKCµ [73], impairs pCREB in fibrocytes but not in fibroblasts. This divergence suggests that the coupling of PKC to CREB may exhibit isoenzyme specificity and that PKCβII may be far more effective than PKCµ as an upstream effector of CREB activation.

IL-6, like other pleiotropic cytokines, is produced widely in response to multiple stimuli [74]–[79]. It supports B cell function by driving immunoglobulin synthesis and promoting plasma cell differentiation [80]. It regulates fat metabolism [21] and has been implicated in inflammatory processes such as those occurring in TAO [81]. Serum levels of IL-6 are elevated in individuals with GD [19], [82] and are particularly high in those with TAO [83]. Moreover, it can be detected in diseased thyroid and its levels in the TAO orbit appear to correlate with tissue expansion [84]. The current observations identify cAMP independent signaling pathways involved in the regulation by TSH of IL-6 in cells playing central roles in orbital GD. It is possible that a component of the involvement of TSHR in the pathogenesis of TAO might be completely independent of its role in provoking the generation of cAMP. Whether the TSHR molecules utilizing separate post-receptor machinery diverge structurally or functionally from those involved in “traditional” signaling dominating the thyroid is uncertain. Similarly, whether the TSIs activating hormone synthesis within the thyroid might be distinguished in any way from those antibodies acting on fibrocytes in the context of the circulation or within the orbit remains to be determined. Absence of the requisite molecular machinery for cAMP generation in fibrocytes suggests that these cells are ideal for examining alternative pathways involved in TSHR signaling. Further, these findings identify a potentially attractive cohort of therapeutic targets for modulating the inflammatory phenotype of fibrocytes.
